# Acceptability of text messages for safety netting patients with low-risk cancer symptoms: a qualitative study

**DOI:** 10.3399/bjgp18X695741

**Published:** 2018-03-27

**Authors:** Yasemin Hirst, Anita Wey Wey Lim

**Affiliations:** Research Department of Behavioural Science and Health, University College London, London.; Centre for Cancer Prevention, Wolfson Institute of Preventive Medicine, Queen Mary University, London.

**Keywords:** early diagnosis, patient safety, primary care, qualitative research, signs and symptoms, text messaging

## Abstract

**Background:**

Safety netting is an important diagnostic strategy for patients presenting to primary care with potential (low-risk) cancer symptoms. Typically, this involves asking patients to return if symptoms persist. However, this relies on patients re-appraising their symptoms and making follow-up appointments, which could contribute to delays in diagnosis. Text messaging is increasingly used in primary care to communicate with patients, and could be used to improve safety netting.

**Aim:**

To explore the acceptability and feasibility of using text messages to safety net patients presenting with low-risk cancer symptoms in GP primary care (txt-netting).

**Design and setting:**

Qualitative focus group and interview study with London-based GPs.

**Method:**

Participants were identified using convenience sampling methods. Five focus groups and two interviews were conducted with 22 GPs between August and December 2016. Sessions were audiorecorded, transcribed verbatim, and analysed using thematic analysis.

**Results:**

GPs were amenable to the concept of using text messages in cancer safety netting, identifying it as an additional tool that could help manage patients and promote symptom awareness. There was wide variation in GP preferences for text message content, and a number of important potential barriers to txt-netting were identified. Concerns were raised about the difficulties of conveying complex safety netting advice within the constraints of a text message, and about confidentiality, widening inequalities, and workload implications.

**Conclusion:**

Text messages were perceived to be an acceptable potential strategy for safety netting patients with low-risk cancer symptoms. Further work is needed to ensure it is cost-effective, user friendly, confidential, and acceptable to patients.

## INTRODUCTION

In the UK, around 90% of cancers present initially to GPs in primary care,^1^ yet almost half of cancers in England are diagnosed at late stages.^2^ The non-specific nature of cancer symptoms, particularly early on in the evolution of malignancy, is a key barrier to early diagnosis.^3^ Symptoms can range in seriousness from self-limiting, requiring no treatment, to indicative of serious underlying illness, such as cancer. However, presenting signs and symptoms are often not of enough concern to prompt referral. Safety netting is an important diagnostic strategy for managing patients with potential cancer symptoms. It encompasses a broad variety of activities that aim to stop patients from ‘slipping through the net’, and avoid misdiagnosis or delayed diagnosis. One of the main safety-netting approaches is to ask patients to return if symptoms persist beyond a given time period.^4^^,^^5^ Patients may also be advised to look out for signs or symptoms that would prompt follow-up or further investigation. Inadequate safety netting has been associated with delays in cancer diagnosis.^6^^,^^7^ Currently, there is no agreement on how to apply safety netting in primary care,^5^ and a recent review highlighted the lack of empirical evidence for safety-netting practices in primary care in relation to cancer diagnosis.^8^ National Institute for Health and Care Excellence (NICE) guidelines for cancer referral^9^ include general recommendations for safety netting, but also acknowledge that these are limited by the lack of empirical evidence.

A weakness of current safety-netting practices is the reliance on patients to re-appraise their symptoms and to make follow-up appointments.^10^ Worry about wasting the doctor’s time and not wanting to bother the doctor are well-documented barriers to help seeking in the UK.^11^ For patients who are given safety-netting advice when they consult, a potential pitfall is that 40–80% of medical information provided by healthcare practitioners is forgotten immediately, and almost half of the information that is remembered is recalled incorrectly.^12^ Evidence also suggests that misunderstanding and miscommunication are commonplace in primary care consultations.^13^

Technology-based interventions, such as text message (or SMS [short message service]) alerting, could potentially help to address these issues, and may be a simple and effective approach to ensuring timely diagnosis.^14^ The delivery of text messages can be automated, and there is increasing evidence that text messages are a useful tool for appointment reminders,^15^ managing chronic illnesses, and smoking cessation.^16^ Mobile phone use is virtually ubiquitous in the UK (93% of adults own a mobile, and 85% of those aged >55 years use text messaging).^17^^,^^18^ Text messages could provide a useful additional platform to engage with patients who require safety netting in primary care. This approach could also address help-seeking barriers by providing an additional nudge and reassurance that the doctor wants to see a patient.

**How this fits in**

Safety netting is an important diagnostic strategy for managing patients with potential cancer symptoms. Asking patients to return if symptoms persist is a common approach, but has inherent weaknesses. Text messages could be a simple, effective, and low-cost way of improving safety netting of patients with low-risk cancer symptoms (txt-netting). This study explored GPs’ views and preferences, and the acceptability of txt-netting. Findings suggest that GPs are amenable to txt-netting in principle, but had wide preferences for text message content, and identified many potential barriers that need consideration before implementation is recommended.

GP involvement in the design and development of these interventions in primary care is crucial to ensure successful implementation.^19^ As part of a feasibility project on safety netting using text messages (henceforth referred to as txt-netting), this focus group study explored GPs’ views on the concept of using text messages to safety net patients with potential cancer symptoms, and its potential applications, barriers, and facilitators.

## METHOD

The authors conducted semi-structured GP focus groups and telephone interviews. First, GPs discussed their safety-netting practices that are in place, followed by discussions to explore the potential use of text messages within GP practices. The authors framed the discussion using a social constructionist perspective to encourage GPs to consider txt-netting in the broader social context of their GP practice (for example, patient population, workload, or operational procedures), as opposed to merely personal views and preferences.

### Data collection

Between May and September 2016 the authors invited GPs to take part in focus groups via clinical commissioning groups, GP mailing lists, and existing contacts. Focus group participants were limited to GPs to ensure the views on the acceptability of text messages to safety net patients with low-risk cancer symptoms reflect the direct users. The authors used a convenience sampling method to allow GPs the flexibility of choosing a focus group according to their availability using online scheduling software (Doodle; http://doodle.com/en_GB/), and to conduct the focus groups in a short period of time. The authors also allowed focus groups to take place during clinical meetings to engage with as many GPs as possible.

Focus groups and the interviews took place between August and December 2016. All focus groups were led by an experienced qualitative researcher, with the second researcher acting as observer. Before the discussions began, GPs completed a demographic questionnaire and signed consent forms. The duration of the focus groups ranged from 60 to 90 minutes. Focus groups and interviews were audiorecorded and transcribed verbatim.

### Topic guide

A semi-structured topic guide (Appendix 1) was developed following a stakeholder meeting with GPs, commissioners, and GP facilitators (that is, individuals who provide training and guidance about new initiatives in primary care). Before recruitment, the topic guide was tested in a mock interview, with a GP checking that the duration was appropriate, and to ensure that all the questions were relevant and would elicit responses that would meet the authors’ objectives. Discussions started by exploring GPs’ understanding of cancer safety netting, and existing safety-netting pathways and procedures (including patient communication). Participants were then asked to discuss the potential role of text messages in safety netting patients with low-risk cancer symptoms, including how and when they could be used. Each group was asked to indicate where on the safety-netting pathways it could be useful to apply identified text messages. GPs were asked to discuss the potential barriers and facilitators of txt-netting.

### Data analysis

The authors analysed the data using an inductive thematic approach.^20^ Both researchers initially read all of the transcripts, and one coded all transcripts. Two of the focus group transcripts were independently coded by the second researcher. Initial codes were grouped into potential themes. A thematic map was produced, and then discussed and agreed by both researchers. Both researchers agreed that the final focus group did not produce new codes and themes, and the themes were also supported by the interviews.

## RESULTS

### Sample characteristics

Overall, 24 GPs agreed to take part in the focus groups. Due to time restrictions two were unable to attend their intended focus group sessions and had telephone interviews instead (both audiorecorded). Groups 2 and 4 each had one participant drop out at the last minute. Most groups were a mixture of GPs from different practices, except groups 4 and 5. In total, 20 GPs attended one of the five discussion groups, with a minimum of two and maximum of seven GPs per group. Almost half of the participants were female, and aged 29–59 years, with an average of 8 years as an active GP; 27% were involved in research, and 55% were employed full time. Table 1 outlines sample characteristics.

**Table 1.** Characteristics of the GP sample

	***n* (%)**
**Total**	22 (100)

**Sex**	
Male	12 (55)
Female	10 (45)

**Ethnicity**	
White British/other white	9 (41)
African/Caribbean	4 (18)
Asian (Indian/Pakistani/Bangladeshi)	8 (36)
Unknown	1 (5)

Mean age (range), years	38.9 (29–59)

Number of years as an active GP (range)	7.71 (1–30)

**Employment status**	
Full time	12 (55)
Part time/salaried	7 (32)
Unknown	3 (13)

**Research active**	
Yes	6 (27)
No	16 (73)

### Potential role of txt-netting

**Figure 1.**
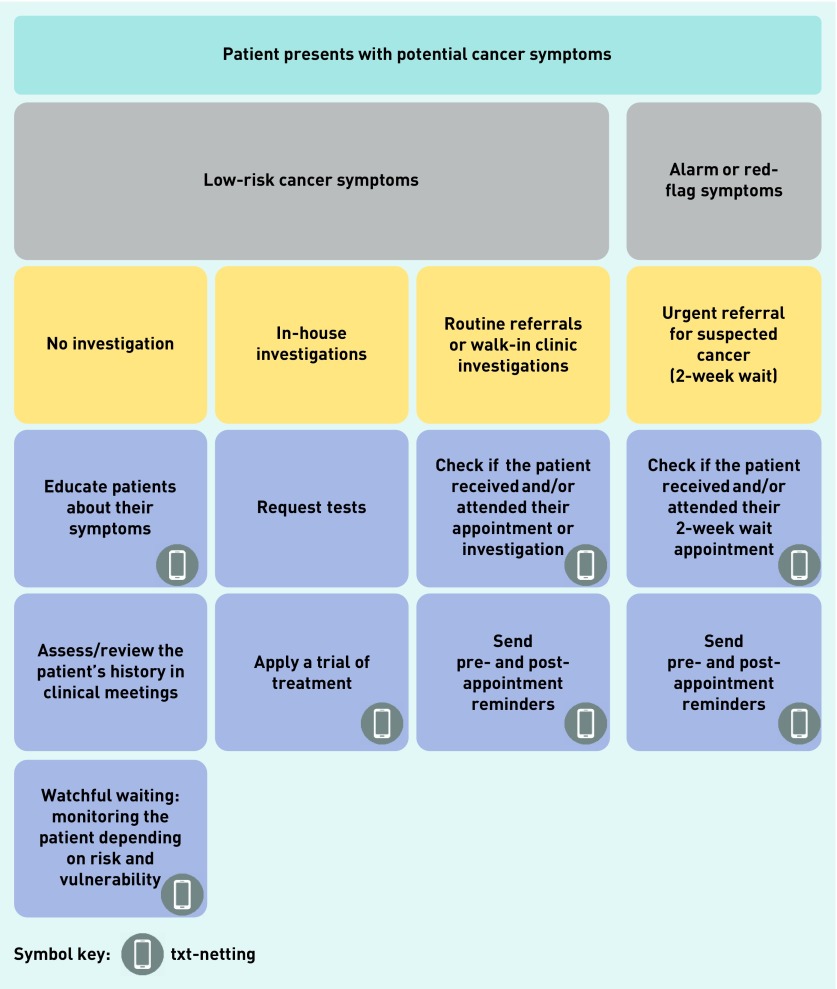
***Potential pathways for use of txt-netting in primary care.***

GPs identified several cancer safety-netting pathways that could benefit from an automated text message (Figure 1). GPs agreed that a key function could be to raise symptom awareness among patients. This would comprise a simple message advising what to look out for, and what actions to take if symptoms persist. For patients with low-risk cancer symptoms (that is, not red-flag symptoms), the purpose of a text message will be to reaffirm what was discussed during the consultation, and to check if symptoms resolve within a given time period (watchful waiting) and/or with treatment:

‘We have different sections in our EMIS. You have problem, you have history, examination … You might have a safety net, and then if that could be automatically incorporated into a text that you send out, then you could write straight into the message what you want to be sent out to the patient. That way you know that what was discussed in the consultation has been reinforced with a text message so the patient can go back and use it as a reference maybe 2 weeks down the line. “Okay, that’s what was discussed. Do I have that? I don’t.”’

(Group 2, male 1)

‘If there’s sort of just one text, one standard text, which you are going to have, then I would probably say “Please book an appointment if you are not better.” Yes. And if you can have another one for “Please remember to have your tests”, that would be good, too.’

(Interview 1, female)

GPs thought that a text message could help improve follow-up processes by rapidly identifying patients who need further assessment. GPs opined that this would be particularly useful when a patient’s symptoms do not warrant urgent referral or immediate investigation, and for patients who are more likely to slip through traditional safety-netting approaches (for example, due to language barriers). They also thought that txt-netting could help to ensure that patients review their symptoms in a timely manner, and would encourage patients to return. However, GPs highlighted that there may still be workload implications for patients requiring follow-up:

‘It would be useful, I think, if it was like: “Yes, it’s resolved”, because then we can kind of close that off. If it’s: “No, it’s not resolved”, then we still need to proactively help them book in somehow, either for a phone follow- up or a clinical follow-up, or whatever.’

(Group 5, male 1)

‘“If your symptoms aren’t better, please book another appointment now”, then that actually would be really handy, because I do think that one of the really difficult things is people can never remember how long they’ve had something.’

(Interview 1, female)

Two further suggested uses of text messages for safety netting that were outside of watchful waiting were identified: to confirm that urgent cancer (2-week wait) referral appointments are received and attended, and to send reminders for walk-in appointments to investigate symptoms (for example, a chest X-ray). Many GPs reported that they currently use manual, paper- based systems, such as personal diaries, to track patients with urgent cancer referrals:

‘If there was just an automated system that would offer a text message if somebody had not attended a 2-week wait appointment, whether it was because they didn’t attend, or whether because the hospital themselves messed up the appointment in some way administratively, or a letter didn’t get through, but just to know that there was an automated text would support me so much in my practice, and quite a proportion of people miss their 2-week wait appointments. It’s shocking.’

(Group 3, male 4)

*‘We have a lot of DNAs, do not attends, in our local hospital, and …* [unclear] *… would be a big proportion of our budget, but also sort of waste the resources, and we get do not attend cancer referrals. So we’ve gone to the effort of thinking this patient has cancer, sent them a letter, but they haven’t attended. We never really find out why that is, but we have to chase them up on that. So it would be great if we had that in a simple text message, I suppose.’*

(Group 4, male 1)

### Potential barriers to txt-netting

#### GP preferences for txt-netting

GPs expressed a wide range of preferences for how txt-netting should be applied, and for text message content. Some wanted to be able to tailor text message content, whereas others preferred generic wording (recognised as more time efficient). Some GPs preferred a patient-managed approach, relying on patients to self-assess and book follow-up appointments, whereas others preferred a GP-managed approach, in which GP practices would contact patients who replied to the text message to confirm ongoing symptoms, that is, two-way messaging:

‘I would prefer to send out the text myself, knowing the patient, and to write what specifically I am concerned about. Because you kind of know what you’re worried about at the end of your consultation, and the specific things to that patient that you’d want to question about.’

(Group 4, male 1)

‘I think if I could generate a text message to remind somebody to do something, or to review somebody … if we could have a little text library, in EMIS, or MJog, whatever it is, and you could choose that text, that text, that text, and they’re standardised texts, so you don’t have GPs writing them. You’ve got to make it quick and easy for GPs, and you’ve got to also be able to make it a handover system, so … there’s no point in GPs spending a lot time doing admin work, they’d probably need to be doing clinical work.’

(Interview 2, female)

GPs also highlighted the need to automate txt-netting to maximise efficiency:

‘I think that would be a huge undertaking to remember to send all those text messages in 2 weeks. I know that I wouldn’t be able to encompass that on my workload. So I think the most efficient way would be, as you close your consultation, that message would go off.’

(Group 1, female 1)

#### Patients’ understanding of text messages

The challenge of accurately conveying safety-netting advice within a single text message was discussed as a key barrier:

‘I question whether or not one generic message would be enough. So it would be wanting them to understand that: “The consultation we had today left me with a bit of concern, so I would like to see you again.”’

(Group 2, female 1)

This led GPs to question whether or not txt-netting would be flexible enough to work in practice, particularly for patients with comorbidities and multiple symptoms. There were concerns that this would lead to issues with patients’ understanding of the text message:

‘We think it’s their haemorrhoid, but we want to make sure that it’s resolved in 2 weeks, and so we waited 2 weeks to ask them: “Have you seen this resolved?”’

(Group 5, female 2)

‘They might be like, “Which one?” They might be thinking about their cough, or …’

(Group 5, female 1)

‘I can see problems. If it’s getting better, is that resolved, or does it have to be completely better? You can see how you could interpret it differently. We know what we mean, but that doesn’t necessarily come across in 144 characters.’

(Group 5, male 3)

#### Timing of text messages

Concerns were also raised about the appropriate timing of text messages, and how this would change depending on the nature of the symptom. GPs also pointed out that timely review could be negated by the time needed to make and receive appointments:

‘Remember, they’re going to get the text in 2 weeks, but actually they’ve got to book an appointment, so it might be another 2 weeks before they actually come in.’

(Group 3, male 3)

### Ethical considerations of txt-netting

Several ethical considerations were raised in relation to txt-netting. These included patients without access to a mobile phone, and the incomplete or inaccurate nature of mobile phone numbers in primary care records. GPs also referred to sub-populations in their practices who could be marginalised by txt-netting (for example, older or tech-novice patients). This was discussed as a potential pitfall of mobile health interventions:

‘Here, people aren’t so … they’re not as phone friendly. They’re not as tech savvy, mobile. I’ve had patients I’m trying to get onto the phone to type in numbers and they’ve been like, “I don’t know how to work my phone.’”

(Group 4, male 2)

All focus group discussions raised the importance of patient consent and confidentiality when sending text messages with potentially sensitive information, such as symptoms:

‘If you’re with your mates in the pub or whatever, and you have your phone there. Suddenly it flashes: “Has your rectal bleeding improved?”’

(Group 5, female 2)

Patient consent to txt-netting was considered by GPs to be essential:

‘If you are sending text messages, that phone may be available to other members of the family. You may need to check with them. Other family members may not be aware about these worrying symptoms that this patient has. They might not want other people to know about it, so do they really want a text that is going to be on the phone that other people can read, potentially? It might pop up when someone else has the phone. You might need to think about whether that’s appropriate.’

(Group 5, female 1)

‘It’s a patient choice if they want to be safety netted. I might see a 90-year-old who has a potential cancer risk. They might not care any more that they have cancer. Why do they need safety netting?’

(Group 2, male 1)

### Attitudes to txt-netting

Although there were concerns about implementation of txt-netting in practice, the use of technology for safety netting in general was welcomed, and was recognised as currently underused in primary care, with many GPs relying on manual, paper-based systems. To some extent, they suggested that text messages are already used to safety net (for example, appointment reminders or reporting of tests results). Txt-netting was perceived as a useful concept worth exploring further, most viewing it as an additional tool for patient management:

‘I think it’s an option we should be using. I think there’s probably a lot of scope within IT that we’re not using. I think with cancer and safety netting, the more systems we have, which we could use, if we need … the more tools we have we can pick up when there’s a problem. If texting is one of them, then that’s great.’

(Interview 2, female)

*‘It’s just another tool in your armoury, I suppose. You can do verbal safety netting, you can do, maybe, written safety netting. Text messaging would just be another tool.*’

(Group 2, male 2)

## DISCUSSION

### Summary

The findings suggest that GPs are amenable in principle to the concept of using text messages to safety net patients with low-risk cancer symptoms (txt-netting). There was wide variation in GP views on how txt-netting could be applied, and on text message content, including additional applications in patients with a higher index of suspicion for cancer. A number of important potential barriers to txt-netting were highlighted that need careful consideration. The key issues identified were around the difficulties of conveying safety-netting advice within the constraints of a text message format, concerns about confidentiality and inequalities, the existing lack of available appointments, and the potential impact on GP workload.

### Strengths and limitations

As far as the authors are aware, GPs’ acceptability and feasibility of primary care-based messages for cancer safety netting have not yet been explored. Thus, having a focus group study with a drip-fed discussion guide enabled interactive and eclectic dialogues about current safety-netting practices in cancer diagnosis, and how txt-netting could be applied in practice. The interaction between GPs allowed the emphasis of the conversation to be on implementation in primary care rather than personal preferences. However, the generalisability of these findings may have been affected by the opportunistic approach to recruitment, the inclusion of only London-based GPs, and self-selection bias.

The authors excluded GP practice administrative staff who are likely to have a key role in txt-netting if implemented. However, the main aim of the study was to explore txt-netting acceptability among GPs as the anticipated main-end users. The authors plan to explore acceptability with patients and other non-clinical stakeholders in a follow-on pilot study that will assess txt-netting in practice.

### Comparison with existing literature

The findings corroborate a previous UK study which found that GPs are generally highly amenable to incorporating new technologies in the routine delivery of primary care, welcoming their ability to enhance practice efficiency.^21^ Interestingly, the same concerns were raised by GPs about the potential for technology and e-health to widen inequalities, increase workload, and raise medicolegal issues. Furthermore, a report commissioned by the Department of Health in England suggests that GP safety-netting practices in cancer diagnosis are extremely variable.^4^

As far as the authors are aware, there is no existing literature that has explored the use of text messages for safety netting patients with potential cancer symptoms. However, a recent UK study^22^ reported large increases in the use of text messages for patient communication in primary care. The same study found that both GPs and patients cited more effective time management as a key benefit of primary care-based text messages and, as with this study, confidentiality was identified as the main risk. Text message reminders have been shown to improve cancer screening uptake.^16^^,^^23^ Also, there is encouraging evidence that text messages can be used in primary care to monitor acute and chronic conditions, and that this reduces the cost of care, improves patient adherence, and promotes continuity of care.^15^ Primary care physicians in a survey study in Switzerland reported that the use of text messages improved patient follow-up.^24^

### Implications for research and practice

There is limited anecdotal evidence that clinicians are already using text messages to offer advice to patients and arrange appointments for further assessment in secondary care.^25^ Txt-netting in its simplest form could be used to enhance what GPs already do ‘intuitively’ in terms of safety netting. Patients have reported that safety-netting advice is often too vague to be useful,^26^ and txt-netting could potentially provide concrete advice for patients to refer back to. However, this study highlighted that even simple txt-netting approaches could have unintended consequences. Particular care needs to be taken with text message content,^27^ patient confidentiality, impact on GP workload, and consideration of how txt-netting may shift responsibility for follow-up from patient to GP, and vice versa. Although there was much discussion around tailored text messages (which is technically possible), this approach may be too complicated or labour intensive to implement in practice.

Further research is needed before widespread implementation of txt-netting in primary care is recommended. The next logical step is a pilot study to assess the feasibility and acceptability of txt-netting in practice, taking into account the potential issues identified in this study. The patient dimension and patient acceptability of txt-netting would also be important to establish. Subsequently, a large-scale implementation study would be required to assess the impact of txt-netting on patient return times, opportunity costs, and cancer diagnosis timelines, and to establish best-practice guidelines. Finally, with the rapid advances in e-health and the use of technology in health care, it is highly likely that, in the future, txt-netting could be further enhanced, for example, by including e-consultations, or linking to phone web-based apps for automated appointment booking.

## Appendix 1. Brief focus group topic guide

**Table:**

*This project is part of a Cancer Research UK innovation grant, and we would like to find out your views on safety netting using text messages. But before we start talking about text messages, and how we can apply them to safety netting, I would like to find out what low-risk-but-not-no-risk of cancer means to you.* 1) What is the challenge for a GP when a patient comes in with a low-risk-but-not-no-risk symptom of cancer? 2) How do you think you apply safety netting for low-risk-but-not-no-risk cancer symptoms? 3) What are the benefits of safety netting in cancer diagnosis? 4) What are the issues/challenges with safety netting in cancer diagnosis? *Current practice* 5) *In what ways do you document safety netting?* 6) *In your opinion what would be the best practice of safety netting for low-risk-but-not-no-risk cancer symptoms, if we had all the resources to facilitate this in practice?* *Patient communication* 7) *How do you inform your patients about your reasons for safety netting at the end of the consultation?* 8) *What is the role of the patient when they are safety netted?* *Txt-netting* *As a part of a feasibility study, we will be using text messages to enhance the ways in which we communicate safety netting with patients. We are hoping to include text messages within the patient pathway after their first consultation, which is concluded with a diagnostic uncertainty and the best action is to safety net this patient.* 9) How do you think we can incorporate text messaging to improve safety netting for low-risk-but-not-no-risk symptoms of cancer, and facilitate early diagnosis? 10) What would be the benefits of safety netting using text messages? 11) Are there any practical barriers/negative implications of implementing this in your practice? 12) How do you think patients would feel about txt-netting? 13) Would you use it? Why? 14) How would you use text messages for safety netting these patients with non-specific/low-risk-but-not-no-risk symptoms of cancer? 15) If we were to implement this in practice, what do you think the messages need to include?
